# Linking health literacy to malaria prevention and treatment behaviours: evidence from a community-based study in Gabon

**DOI:** 10.1186/s12913-026-15138-1

**Published:** 2026-08-24

**Authors:** Caroline Namubiru, Saidou Mahmoudou, Ynous Djida, Bertrand Lell

**Affiliations:** 1 Department of Economics, Faculty of Economics and Social Sciences, University of Tübingen, Tübingen, Germany; 2 Centre de Recherches Médicales de Lambaréné (CERMEL), Lambaréné, Gabon; 3 Department of Medicine I, Division of Infectious Diseases and Tropical Medicine, Medical University of Vienna, Vienna, Austria

**Keywords:** Health literacy, Malaria, Preventive behaviour

## Abstract

**Background:**

Health literacy (HL) affects how individuals engage in preventive behaviours, seek prompt treatment, select appropriate malaria prevention or treatment options, adhere to care and act on health information. Little is known about its role in malaria prevention and treatment behaviours. Therefore, this study investigated the extent to which HL relates to malaria health behaviours.

**Methods:**

A cross-sectional household survey across urban and rural areas of Gabon using a sample of 504 respondents. HL was measured using the French HLS-EU-Q16 and scored as well as categorised as inadequate, problematic, or sufficient. Malaria health behaviours included regular utilisation of bed nets, uptake of indoor residual spraying (IRS), treatment-seeking for illness, medication adherence, bush clearing, asking medical practitioners questions and Dosage knowledge. The HL scale was first assessed for psychometric suitability within the study sample before conducting subsequent regression analyses. Multivariable logistic regression models were employed to assess associations between HL and malaria-related behaviours, with adjustment for demographic, socioeconomic, and health-environment factors.

**Results:**

59.7 per cent of respondents demonstrated sufficient HL with higher proportions among those with at least secondary education (above 65%), urban residents (61.4%), those aged between 25 and 34 years of age (66.4%) and those using formal health information sources (60.2%). Significant bivariate associations were revealed between HL and dosing knowledge, medication adherence, prompt treatment seeking and asking questions. No bivariate associations were observed for Bednet use or bush clearing. However, multivariate logistic regression adjusting for demographic, socio-economics and health environment factors revealed significant associations of HL with dosing knowledge (AME = 0.022, *p* < 0.001), medication adherence (AME = 0.013, *p* < 0.01; OR = 1.089), asking questions (AME = 0.018, *p* < 0.001; OR = 1.092), and bednet use (AME = 0.012, *p* < 0.05). Treatment-seeking behaviours, bush clearing and indoor spraying were not significantly associated with HL (*p* > 0.05).

**Conclusion:**

HL has associations with knowledge-dependent and communicative aspects of malaria management. Therefore, literacy-focused interventions in malaria-endemic regions should be prioritised alongside structural approaches addressing net access and healthcare availability to maximise impact on malaria-related outcomes.

**Clinical trial number:**

Not applicable.

**Supplementary Information:**

The online version contains supplementary material available at 10.1186/s12913-026-15138-1.

## Introduction

Malaria is a leading public health challenge across sub-Saharan Africa, with profound implications for morbidity, mortality, and socioeconomic development. Despite significant efforts to eliminate and control malaria, the disease burden persists. Gabon bears a substantial malaria burden, with the disease ranking among the leading causes of morbidity and mortality, particularly among children under five years of age and pregnant women. The country experiences perennial malaria transmission facilitated by its equatorial climate, which provides optimal breeding conditions for mosquito vectors [1]. Health literacy, defined as the ability to access, understand, appraise, and apply health information, is a key determinant of health behaviours and service utilisation [2–4]. The HLS-EU-Q16 has been widely applied to assess HL and study its relationship with health behaviours [5–7]. Low HL can limit comprehension of preventive measures, delay treatment-seeking, and reduce adherence to medical guidance. Studies from several European populations have demonstrated associations between lower HL and unhealthy behaviours, including physical inactivity, poor dietary practices, and increased smoking and alcohol consumption, though the strength of these associations varies by specific behaviour and population [8–11]. Research on HL and health behaviours has predominantly focused on non-communicable disease prevention, including lifestyle and nutrition-related behaviours [3, 12].

Considerably less attention has been devoted to HL’s role in infectious disease prevention, particularly malaria. The skills encompassed by HL are theoretically relevant to behaviours related to malaria prevention and treatment. Understanding prevention messages about bed net use, correctly interpreting treatment instructions, evaluating information about malaria symptoms, and making informed decisions about when to seek care all require adequate HL. However, empirical evidence documenting these relationships in malaria-endemic regions remains limited [13]. Emerging evidence from sub-Saharan Africa suggests that HL plays an important role in malaria control efforts. In Ethiopia, malaria HL was identified as both a barrier and facilitator to insecticide-treated bed net utilisation and prompt diagnosis and treatment, with individuals with higher malaria HL having better adherence to malaria prevention measures [14]. A systematic review of health education interventions across sub-Saharan Africa found that health education significantly improved both malaria knowledge and insecticide-treated net usage [15]. In Ghana, HL interventions using co-created educational materials, including interactive board games and brochures, improved caregiver-health provider relationships and enhanced participatory approaches to malaria management among caregivers of children under five [16].

A recent study in Ghana found that caregivers with high HL incurred higher household costs for managing malaria in children under five than those with lower HL, possibly reflecting more proactive healthcare-seeking behaviour and use of formal health services [17]. However, barriers to effective malaria management persist across many SSA countries, including misconceptions about disease causation, reliance on informal and traditional treatment sources as first-line care, and structural barriers such as distance to health facilities and medication costs [18–20]. A study conducted in Nigeria established that even in educated, semi-urban communities, high awareness does not necessarily translate into appropriate preventive behaviour or formal health-seeking [21]. Understanding the human response to malaria and control programmes has been recognised as crucial to the success of malaria elimination strategies [22]. Health-seeking behaviour, treatment adherence, and the adoption of preventive measures are influenced by individuals’ ability to access, understand, and act on health information. Despite this recognition, the specific contribution of HL to malaria-related behaviours has not been systematically assessed in Central African contexts. Gabon’s unique position with a persistent malaria burden provides an important setting to investigate these relationships. Therefore, this study aimed to: (1) assess the psychometric performance of the French HLS-EU-Q16 within this Gabonese sample, and (2) examine associations between HL and malaria-related prevention and treatment behaviours, including bed net use, treatment-seeking patterns, dosage knowledge, medication adherence, and engagement with healthcare providers.

## Methods

### Study design and setting

We conducted a cross-sectional household survey using quantitative research methods from June to July 2025 in the Moyen-Ogoueé, Ngounié, and Estuaire Provinces of Gabon, encompassing both urban and rural contexts. Lambaréné, Fourplace and Sindara were the main areas of data collection. Data collection was through face-to-face surveys. The study was reviewed by the Institutional Scientific Review Board (SRC) and approved by the Institutional Ethics Committee of the Centre de Recherches Médicales de Lambaréné **(**CERMEL) (2024-04). The target population consisted of Gabonese residents aged 15 years and older.

### Sample size determination and allocation

Household enumeration data from SUDESA (the French version of the Health and Demographic Surveillance System (HDSS)) supplemented by aerial map analysis identified about 5,800 households across the three study areas: Lambaréné (about 5,457 households), Fourplace (80 households), and Sindara (263 households) [23]. The household counts for Fourplace and Sindara were based on complete village registries, while the Lambaréné estimate was derived from SUDESA records and aerial mapping due to the absence of a comprehensive household registry. To determine the sample size, Cochran’s formula [24] for sample size determination, adjusted with the finite population correction (FPC), was used. The formula is given as:

$$\:{n}_{0}={{Z}_{\raisebox{1ex}{$\alpha\:$}\!\left/\:\!\raisebox{-1ex}{$2$}\right.}}^{2}*\frac{pq}{{e}^{2}}$$

where $$\:{n}_{0}$$ denotes the initial sample size, $$\:{Z}_{\raisebox{1ex}{$\alpha\:$}\!\left/\:\!\raisebox{-1ex}{$2$}\right.}$$ is the reliability coefficient of standard error at 5% level of significance = 1.96, p= proportion estimate of the prevalence of malaria equal to 19% [25], and e refers to the level of standard error tolerated/ precision (5%). Therefore, the minimum sample size for the study is 297 households after adjusting for missing data and non-responsiveness by 20 per cent [26, 27].

Given the large differences in population size across the three study areas, proportional allocation would lead to insufficient sample sizes in Fourplace and Sindara [28]. Hence, to address this imbalance and ensure sufficient statistical precision, power allocation was applied as proposed by [29]. This method adjusts sample sizes using a power coefficient (α) between 0 and 1, allowing a compromise between proportional and equal allocation. A value of the power coefficient of 0.5 was selected as it lies between proportional and equal allocation. Sample sizes for each area were then computed based on the formula:

$$\:{n}_{i}=n*\frac{{N}_{i}^{\alpha\:}}{\sum\:{N}_{i}^{\alpha\:}}$$

where $\:{n}_{i}$ is the sample size for each area, * n* is the minimum sample size for the study, $\:{N}_{i}$ is the population size for each area, and $\:\alpha\:$ the power coefficient = 0.5. The minimum final sample sizes for the study areas were then Lambaréné- 221 households, Fourplace- 27 households and Sindara-49 households.

### Household selection

A multi-stage sampling approach was employed. In Fourplace and Sindara, where complete household registries were available from SUDESA, systematic probability sampling was implemented. Households were selected using equal probability intervals, with the starting point chosen randomly. Within each sampled household, available eligible members were invited to participate. In Lambaréné, because there was no existing comprehensive sampling frame, we implemented stratified quota sampling using the SUDESA building maps from CERMEL as a geographic reference. Lambaréné was subdivided into 13 distinct neighbourhoods (referred to as first districts) named: Abongo, Dakar, Grand Village I, Sainte Therese, Adouma, Chateau, Grand Village II, Faisceaux, Lalala, Moussamoukougou, Point V, Atongowanga and City Centre. To ensure geographic representation, we allocated neighborhood-specific target sample sizes (20 to 30 households per neighborhood) proportional to estimated building densities derived from the aerial mapping. Within each neighbourhood, systematic door-to-door visits along predetermined routes were used to select households to achieve neighbourhood-specific targets. Data was collected using a questionnaire, and for each person, a single trained interviewer conducted the interview. In households with multiple eligible members, the study protocol aimed to recruit up to two individuals per household. However, in practice, most households (approximately 95%) contributed only one participant due to availability of household members and participant willingness. Only 13 households contributed two participants, resulting in a sample of 491 unique households. For households where two members participated, interviews were conducted individually and in private to prevent response bias. The final sample comprised 504 respondents distributed as follows: Lambaréné 368 (73%), Fourplace 37 (7.3%), Sindara 62 (12.3%), and Medang Nkoghe 37 (7.3%). Medang Nkoghe, an additional rural village in Moyen-Ogooué province, was included during data collection to strengthen rural representation.

All participants were provided with written information regarding the study before their participation, and they signed consents to participate in the study. Therefore, they were aware of the study objectives, the voluntary nature of participation, the potential risks, and the confidentiality of their responses. Data was collected anonymously, and no personal information was recorded. Additionally, no economic compensation was provided for their participation in the study. Concerning individuals under 18 who completed the questionnaire, prior consent from their parents or representatives was obtained. The interview was conducted in French, the official language in Gabon.

### Data collection instrument

A questionnaire was the data collection instrument, and data were collected on various characteristics including demographic; socioeconomic; health environment; knowledge about Malaria; HL and malaria control behaviours. Questions for HL were adapted from the European Health Literacy Survey Questionnaire, short version comprising 16 items (HLS-EU-Q16 French version) [30] as developed by the HLS-EU Consortium [31, 32]. Although the lengthier version of HLS-EU-Q comprising of 47 items of HL skills may provide optimal validity and reliability, a shorter version enhances the feasibility of measuring HL as part of a large-scale surveillance instrument [33]. The French version of HLS-EU-Q16 was translated from the validated English questionnaire HLS-EU-Q16 and tested for psychometric properties to enable its use in surveys of HL for the general population [30]. This version also captures aspects on health care, disease prevention and health promotion and all features related to accessing or obtaining information relevant to health, understanding information relevant to health, processing or appraising information relevant to health and applying or using information relevant to health. French serves as the official language of Gabon; this existing French version facilitates implementation while maintaining the psychometric integrity of the original instrument. Further, the utility of the HLS for African populations has been demonstrated through its successful cultural adaptation and validation in Ghana, where researchers have developed a culturally adapted version in the Ghanaian language (Akan; Asante Twi) [34] and Cameroon where it was adapted for both English and French speaking populations [5]. The psychometric performance of this version within the present sample is reported in the results section of this study.

There were eight malaria-related health behaviour questions. Four of these were assessed on a five-point scale. For “use of bed nets,” “seeking care when ill,” “seeking care or family or friends when ill,” and “completing prescribed medication,” responses ranged from “Always” (score 4) to “Never” (score 0). Three behaviours were assessed with yes/no/not sure responses: “participation in indoor residual spraying,” “clearing bushes around the home,” and “knowledge of correct dosing when prescribed anti-malarial treatment.” For these, “Yes” was scored 1, “Not sure”, and “No” scored 0. The “Not sure” and “No” responses were scored together because the “Not sure” responses were sparse, which would have produced small or empty cells when cross-classified with the outcome variables and yielded unstable estimates, and the substantive consideration that an uncertain response does not constitute affirmative knowledge or behaviour and is therefore conceptually closer to “No” than to “Yes”. Collapsing on these grounds is consistent with established guidance on handling sparse categorical data in regression [35, 36]. The final question asked about “asking health practitioners questions about malaria or other illnesses,” and was also scored on the same five-point scale from “Always” (4) to “Never” (0). These outcomes are in Section F of the survey instrument and operationalised as follows. Bed net use was measured by asking respondents how frequently they used mosquito nets as a preventive measure against malaria. Participation in indoor residual spraying and environmental clearance around the home were assessed by asking whether respondents participated in household insecticide spraying and vegetation clearance, respectively. Prompt treatment-seeking for oneself and for a household member were assessed by asking how consistently respondents sought immediate care upon experiencing fever or cough or facilitated equivalent care for a household member. These items measure treatment-seeking for symptoms suggestive of malaria rather than for parasitologically confirmed malaria, consistent with standard malaria treatment-seeking indicators used in the Demographic and Health Surveys, which use fever in the preceding two weeks as the accepted proxy for suspected malaria [37]. Cough was included alongside fever because febrile illness in this malaria-endemic setting is rarely symptomatically isolated. The implications of this proxy-based operationalisation are considered in the Limitations.

Dosing knowledge was assessed by asking whether respondents correctly understood a prescription. Medication adherence was measured by asking whether respondents completed their full prescribed course of antimalarial or other treatment even after symptoms resolved. Asking healthcare providers questions captured how consistently respondents sought clarification or additional information from a physician or healthcare professional regarding malaria or other illnesses. Content and cultural relevance of the questionnaire was done before data collection where a panel of stakeholders, including public health experts, sociologists, and health workers, reviewed all items for clarity, objectivity and cultural appropriateness. A pilot study was done with a small group of participants (20) from the target population to evaluate acceptability and ease of response. The questions were clear and well understood and no wording adjustments were made.

### Statistical analysis

First descriptive analyses of the different variables were done using frequency tables and summary statistics where applicable. Whereas the HLS-EU-Q16 French version has already been validated and tested for psychometric properties [30], it has not been tested in Gabon. We employed a comprehensive psychometric validation approach using complementary methods that assess different aspects of scale performance. Internal consistency analysis (Cronbach’s alpha and McDonald’s omega) evaluated the reliability of the scale by assessing whether items measure a cohesive construct, considering values above or equal to 0.70 as satisfactory [38]. Floor and ceiling effects were evaluated by calculating the percentage of respondents achieving the minimum possible score (floor effect) and maximum possible score (ceiling effect). This was done to ensure the scale adequately discriminated among respondents across its full measurement range. Exploratory Factor analysis was assessed to determine the factor loading of each item to the HLS-EU-Q16 item after assessing the existence of interrelation in the data using the Kaiser-Meyer-Olkin (KMO) method and Bartlett’s test of sphericity, where values greater or equal to 0.80 for KMO and significance levels *p* < 0.05 for Bartlett’s test [39]. Exploratory factor analysis was conducted using principal component analysis to assess the dimensional structure of the scale.

Structural validity was assessed using Rasch analysis with dichotomised items (the “very easy” and “easy” categories were merged, and the “difficult” and “very difficult” categories were merged). The Rasch model was fitted using conditional maximum likelihood estimation [40, 41]. The separation reliability indices, person and item separation reliability and differential item functioning (DIF) were estimated using a joint maximum likelihood (JML) Rasch model in R, reported in the Supplementary Material. In addition, Local dependence between items was evaluated using residual correlations, with values exceeding 0.40 considered indicative of substantial dependency [42]. We also assessed known-groups validity by examining whether the HLS-EU-Q16 discriminates between groups expected to differ in HL using both categorical (Chi-square tests) and continuous (Kruskal-Wallis tests) analytical approaches [43, 44]. Based on existing literature documenting strong associations between HL and education [8, 45], and urban-rural residence [46, 47], we hypothesised that HL would show significant differences across these characteristics. Conversely, given the inconsistent or minimal gender differences observed in European HL studies [48], we hypothesised that there would be no significant sex differences. Kruskal-Wallis tests were used rather than parametric alternatives because the HL scores were non-normal as seen in supplementary figure S1.

Associations between HL and malaria-related behaviours were examined using two complementary approaches [49]. First, the behaviours measured on five-point Likert scales (bednet use, prompt treatment-seeking for self, prompt treatment-seeking for family, medication adherence, asking questions) were analysed as ordered categorical outcomes using ordered logistic regression, preserving the complete ordinal structure (Always/ Often/ Sometimes/ Rarely/ Never). Second, these same behaviours were dichotomised (consistent behaviour-defined as Yes: Always/Often = 1; inconsistent behaviour-defined as no: Sometimes/Rarely/Never = 0) and analysed using binary logistic regression. The three behaviours, originally measured dichotomously (indoor spraying, bush clearing, dosing knowledge: Yes/No), were analysed only using binary logistic regression. Both analytical approaches (ordered and binary) were applied to the Likert-scale behaviours to assess whether associations with HL were robust to measurement strategy. Results are reported as average marginal effects (AMEs) for binary outcomes, representing percentage-point changes in predicted probability per one-unit increase in the HL score [50], and as Odds ratios for ordered outcomes. We estimated AMEs, also termed average predicted probabilities, to quantify the relationship between HL and each malaria-prevention behaviour [51]. These estimates represent the average change in the predicted probability of engaging in each behaviour for a one-unit increase in HL score, holding other covariates constant.

The regression models were adjusted to control for relevant covariates. These were selected based on two complementary frameworks that together capture both the structural conditions shaping health behaviour and the individual capacities through which health information is acted upon. The Social Determinants of Health (SDOH) framework [52], guided the inclusion of structural and contextual factors known to influence health outcomes, such as education, employment, residence, health insurance status, and proximity to health facilities, that shape the conditions under which individuals access and respond to health information. The Health Literacy Skills Framework [53] complemented this by directing attention to the inclusion of variables related to the acquisition, processing, and application of health information, such as the source of health information. Specifically, we controlled for age, sex, education level, employment status, residence, health insurance status, proximity to health facilities, availability of community health resources, source of health information, and recent malaria exposure (assessed as “Have you or any member of your family had a malaria experience in the past 2 weeks?”, with response options Yes/No/Not sure). All models were estimated with robust standard errors. Multicollinearity was assessed using both the Variance Inflation Factor (VIF) and the Generalized Variance Inflation Factor (GVIF) [54]. This approach is appropriate because the predictor set includes both continuous variables and categorical variables with multiple degrees of freedom, where standard VIF is not sufficient. There was no evidence of collinearity among the explanatory variables (Mean VIF = 1.32, Maximum VIF = 1.69, Minimum = 1.08. Inclusion of education in the models, the third models in the Tables 7 and 8 show no significant multicollinearity in the model with VIF values of Mean VIF = 2.50, Maximum VIF = 9.91 for secondary level of education, Minimum = 1.05. This indicates that multicollinearity is not a concern and that coefficient estimates are stable with respect to linear dependence among predictors. Similarly, the Generalized variance inflation factors (GVIF) ranged from 1.02 to 1.30, well below conventional thresholds of concern, indicating stable model estimation. Statistical analyses were conducted using Stata version 19 and RStudio.

## Results

Table 1 presents the characteristics of the study participants. 504 respondents completed the questionnaire. The highest proportion of respondents were aged between 25 and 34 years (26.0%). Slightly over half of the respondents were female (51.4%). Almost seven in every ten respondents had attended some level of secondary education (67.9%). As far as occupational status was concerned, 58.5% of respondents were working, 79.8% are insured. Majority of the respondents demonstrated strong knowledge of common malaria symptoms, with fever (89.5%), headache (86.3%), muscle pain (82.9%), and fatigue (79.2%) being the most frequently mentioned, while less than two thirds identified loss of appetite (58.3%). The highest percent of responses for knowledge of prevention methods was for sleeping under bed nets (96.2%), followed by mosquito repellents (67.9%) and eliminating breeding sites (62.1%), while half mentioned taking anti-malarial medication (50.2%). Most of the respondents have sufficient HL (59.7%). The HL score ranging from 0 to 16 had a mean of 12.2 and a standard deviation of 4.2. The median score is 14 which indicates a left-skewed distribution concentrated toward higher literacy levels.

**Table 1:** Characteristics of respondents

Characteristic	*n*	Percent (%)
**Education (%)**		
None	15	2.98
Primary	89	17.7
Secondary	342	67.9
Tertiary	57	11.3
**Sex**		
Female	259	51.4
Male	243	48.2
**Residence**		
Urban	368	73.0
Rural	136	27.0
**Employment**		
Not working	208	41.3
Working	295	58.5
**Age**		
15–24	102	20.2
25–34	131	26.0
35–44	119	23.6
45–54	78	15.5
55+	74	14.7
**Source of Health Information**		
No formal sources	24	4.8
Uses formal sources	480	95.2
**Recent Malaria Exposure**		
No exposure	342	67.9
Recent exposure	161	31.9
**Insurance Status**		
Uninsured	102	20.2
Insured	402	79.8
**Proximity to Health centres**		
Less than 1 km	92	18.3
1 km to 3 km	130	25.8
3 km to 5 km	94	18.7
Above 5 km	174	34.5
**Knowledge of malaria symptoms**		
Fever	451	89.5
Headache	435	86.3
Muscle Pain	418	82.9
Fatigue	399	79.2
Loss of Appetite	294	58.3
Diarrhoea	69	13.7
Other Symptoms	42	8.3
**Knowledge of malaria Prevention methods**		
Sleeping under bed nets	485	96.2
Using Mosquito repellents	342	67.9
Taking anti-malarial medication	253	50.2
Eliminating mosquito breeding sites	313	62.1
Others	6	1.2
**HL level**		
Inadequate	86	17.1
Problematic	117	23.2
Sufficient	301	59.7

**Literacy score** (mean = 12.2; std.dev = 4.2; median = 14; min = 0; max = 16

Note: Variations in totals due to missing values

### Assessment of psychometric properties of HLS-EU-Q16 in Gabon

In the analysis, the 16 questions with answers on a 4-point Likert-scale (1 = ‘very difficult’, 2 = ‘fairly difficult’, 3 = ‘fairly easy’ and 4 = ‘very easy’) were dichotomised for each item: ‘fairly easy’ and ‘very easy’ (1 point) and ‘fairly difficult’ and ‘very difficult’ (0 points). Two sum scores were calculated, one for the original Likert scale (16 to 64 points) and the other for dichotomised items (0 to 16 points), where a higher score corresponds to better HL. The total score was then categorised into “sufficient” (13 to 16 points), “problematic” (9 to 12 points), and inadequate (0 to 8 points) [30].

### Floor and ceiling effects

For the 4-point Likert scale items, fourteen respondents (2.78%) obtained the lowest possible score of 16, while two respondents (0.40%) achieved the maximum score of 64. As both percentages fall well below the commonly accepted 15% cutoff, neither floor nor ceiling effects were present. However, for the dichotomised scale, a ceiling effect was observed with 129 respondents (25.6%) scoring 16, the maximum score but no floor effect was observed as only 2.78% of the respondents scored the minimum. When converted into the three levels of HL, most of the respondents (59.7%) attained a sufficient level of HL, 23.2% a problematic level, and 17.1% an inadequate level of HL.

### Distribution of health literacy responses

Table 2 shows the responses for the individual items of the HLS-EU-Q16. Over two-thirds found it easy to understand doctor’s explanations (68.2%), follow instructions on prescribed medicines (72.6%), make decisions based on doctor guidance (73.5%) and understand advice on health from family members or friends (70.5%). Mean scores for these tasks ranged from 1.91 to 2.09, indicating overall ease. In contrast, accessing and applying information on mental health and health-screening was more challenging. Less than half found it easy or very easy to find information on managing mental health (44.7% with an average of 2.63) and slightly more than half of the respondents understood why health screenings are needed (55.7%; average of 2.42). Judging the reliability of health information in the media and deciding how to protect oneself based on media sources were also moderately difficult (mean 2.24–2.30).

**Table 2:** Distribution of responses to the HLS-EU-Q16

On a scale from very easy to very difficult, how easy would you say it is to… (%)	Very easy	Easy	Difficult	Very difficult	Mean	SD
1. Find information on treatments of illnesses that concern you?	20.23	61.09	16.15	2.53	2.01	0.68
2. Find out where to get professional help when you are ill?	20.62	59.34	17.32	2.72	2.02	0.70
3. Understand what your doctor says to you?	17.54	68.23	12.87	1.36	1.98	0.60
4. Understand your doctor’s or pharmacist’s instruction on how to take a prescribed medicine?	18.45	72.62	7.96	0.97	1.91	0.54
5. Judge when you may need to get a second opinion from another doctor?	12.43	60.97	24.27	2.33	2.17	0.66
6. Use information the doctor gives you to make decisions about your illness?	13.23	73.54	12.06	1.17	2.01	0.55
7. Follow instructions from your doctor or pharmacist?	18.91	72.32	7.8	0.97	1.91	0.55
8. Find information on how to manage mental health problems like stress or depression?	7.39	37.35	39.88	15.37	2.63	0.83
9. Understand health warnings about behavior such as smoking, low physical activity and drinking too much?	18.52	59.06	19.69	2.73	2.07	0.70
10. Understand why you need health screenings?	10.55	45.12	35.94	8.4	2.42	0.79
11. Judge if the information on health risks in the media is reliable?	9.59	54.79	31.31	4.31	2.30	0.70
12. Decide how you can protect yourself from illness based on information in the media?	10.51	58.95	27.04	3.5	2.24	0.68
13. Find out about activities that are good for your mental well-being?	13.4	65.24	18.64	2.72	2.11	0.65
14. Understand advice on health from family members or friends?	11.07	70.49	17.09	1.36	2.09	0.57
15. Understand information in the media on how to get healthier?	10.87	66.8	18.64	3.69	2.15	0.65
16. Judge which everyday behavior is related to your health?	9.55	67.64	19.69	3.12	2.16	0.63

### Validity analysis

Two analyses were conducted to assess the validity of the HL scale in this sample. First, Mokken scaling analysis was performed to verify the assumptions of unidimensionality, local independence, and monotonicity. Loevinger’s H coefficients all exceeded 0.3 indicating acceptable scalability and supporting the formation of a cumulative unidimensional scale [55] in Table 3. Assessment of local dependence revealed that most item pairs demonstrated adequate independence (residual correlations below 0.30). Four item pairs showed slightly elevated correlations ranging from 0.42 to 0.44 (items 1–2, items 11–12, items 8–15 and items 15–16), suggesting minimal local dependence. Second, a Rasch model was fitted using conditional maximum likelihood (CML) estimation to estimate item difficulty and assess fit (Table 4).

**Table 3:** Mokken scalability (Loevinger’s H) for the health literacy scale

Item	Loevinger H coeff
1. Find information on treatments of illnesses that concern you?	0.42
2. Find out where to get professional help when you are ill?	0.50
3. Understand what your doctor says to you?	0.53
4. Understand your doctor’s or pharmacist’s instruction on how to take a prescribed medicine?	0.69
5. Judge when you may need to get a second opinion from another doctor?	0.49
6. Use information the doctor gives you to make decisions about your illness?	0.56
7. Follow instructions from your doctor or pharmacist?	0.73
8. Find information on how to manage mental health problems like stress or depression?	0.64
9. Understand health warnings about behavior such as smoking, low physical activity and drinking too much?	0.51
10. Understand why you need health screenings?	0.59
11. Judge if the information on health risks in the media is reliable?	0.60
12. Decide how you can protect yourself from illness based on information in the media?	0.58
13. Find out about activities that are good for your mental well-being?	0.54
14. Understand advice on health from family members or friends?	0.46
15. Understand information in the media on how to get healthier?	0.56
16. Judge which everyday behavior is related to your health?	0.51

Item difficulty parameters spanned a wide range, from − 1.98 logits (item 7, “follow instructions from your doctor or pharmacist”) to + 2.48 logits (item 8, “find information on how to manage mental health problems”), indicating good coverage of the HL continuum (Table 4). Healthcare interaction tasks were the easiest items to endorse, including following instructions from a doctor or pharmacist (-1.98), understanding prescription instructions (-1.94), using the doctor’s information to make decisions (-1.13), and understanding what the doctor says (-1.01). In contrast, health-promotion and information-appraisal tasks were the most difficult, notably finding information on managing mental health problems (2.48), understanding the need for health screenings (1.71), and judging the reliability of media-based health information (1.08). This gradient indicates that respondents possessed relatively strong functional HL for direct clinical encounters but weaker critical and communicative HL in health-promotion contexts.

Item-level fit was acceptable for most items, with standardised infit and outfit statistics falling within the ± 2.5 range. Three items showed statistically significant departures from model expectation. Item 1 (“find information on treatments”) underfitted the model, with both outfit (Z = 2.89) and infit (Z = 2.87) exceeding the upper bound, indicating more unexpected response variation than the model predicts. Item 15 (“understand media health information”) showed overfit (infit Z = -2.56), indicating responses that were more predictable than expected. Item 14 showed a significant item-trait interaction (*p* = 0.001) although its fit statistics remained within bounds. The remaining 13 items demonstrated satisfactory fit. The global item-trait interaction test (R1c = 304.98, df = 210, *p* < 0.001) and the Andersen likelihood ratio test (LR = 319.29, *p* < 0.001) were statistically significant, which would conventionally indicate departure from strict Rasch expectations and non-invariance of item difficulties across ability strata. However, these global tests must be interpreted with substantial caution in the present sample. The distribution of total scores was markedly concentrated toward the upper end of the scale given the negative skewness of the HL scale while the lower score groups were sparsely populated. The estimation routine itself flagged this condition, noting that score groups with fewer than 30 individuals may render the fit tests invalid. Because the chi-square approximations underlying both the R1c and Andersen tests assume adequate cell sizes, the statistically significant global fit results may substantially reflect this sparseness in the lower score strata rather than genuine misfit of the scale. Person and item separation reliability were 0.745 and 0.984, respectively, indicating acceptable reliability for distinguishing respondents with different levels of HL and excellent precision in estimating item locations along the latent continuum. Differential item functioning (DIF) was assessed by sex, age group, and education level using logistic regression. No meaningful DIF was detected by sex: although three items reached nominal significance, all had negligible effect sizes (Nagelkerke R² < 0.02) and none survived correction for multiple comparisons, indicating the scale functions equivalently across men and women. By age and education, a small number of items showed statistically significant DIF. However, only two that is understanding a doctor’s or pharmacist’s instructions (item 4) and following those instructions (item 7), reached a meaningful effect size, and did so consistently across both characteristics. The remaining flagged items showed negligible effects. Full item-level DIF results are reported in Supplementary Tables (S3–S5).

**Table 4:** Item difficulty and fit statistics from the conditional maximum likelihood (CML) Rasch model for health literacy

Item	Difficulty	SE	Outfit (Z)	Infit (Z)	*p*-value
**1**	-0.43	0.20	2.891	2.867	**0.000**
2	-0.30	0.20	0.956	0.304	0.613
3	-1.01	0.22	-0.172	0.600	0.207
4	-1.94	0.26	-1.031	-1.397	0.303
5	0.33	0.19	0.812	1.210	0.510
6	-1.13	0.22	-0.966	0.103	0.559
7	-1.98	0.26	-1.315	-2.240	0.058
8	2.48	0.19	0.714	1.617	0.353
9	-0.06	0.20	-1.091	-0.316	0.616
10	1.71	0.18	0.216	1.531	0.117
11	1.08	0.18	-1.292	-2.245	0.168
12	0.69	0.19	-1.748	-1.908	0.170
13	-0.14	0.20	-1.810	-1.769	0.731
**14**	-0.48	0.20	1.502	1.805	**0.001**
**15**	-0.04	0.20	-2.448	-2.562	**0.002**
16 ᵃ	0.00		-1.167	-0.341	0.596

Note. Item difficulty and fit statistics were estimated using a dichotomous Rasch model with conditional maximum likelihood (CML). Outfit and Infit are standardised (Z) fit statistics; values within ± 2.5 indicate acceptable fit. Items shown in bold (1, 14, 15) showed statistically significant item-trait interaction (*p* < 0.05). ᵃ The difficulty of item 16 was fixed at 0 as the reference. Global fit: R1c = 304.98, df = 210, *p* < 0.001; Andersen LR = 319.29, *p* < 0.001.

### Construct validity: known-groups method

The analyses of relationships between HL levels and different study characteristics (age, education, sex, residence, employment, and sources of health information) provide findings in Table 5. Statistically significant associations were observed for education (*p* < 0.001 for both chi-square and Kruskal-Wallis tests), residence (*p* < 0.05 for Kruskal-Wallis tests), and use of formal health information sources (*p* < 0.05 for both tests). No significant associations were observed for sex. Employment was significantly associated with categorised HL levels on the chi-square test (*p* < 0.001) but not on the Kruskal-Wallis test of the continuous score (*p* = 0.426). Inadequate HL was 40 per cent among those with no education compared with 10.5 per cent among those with tertiary education, was higher in rural areas (23.5%) than urban areas (14.7%) and was more prevalent among those without formal information sources (37.5%) versus those with formal sources (15.8%).

**Table 5:** Differences in health literacy by various characteristics

Characteristics	Inadequate HL	Problematic HL	Sufficient HL	Chi2	Chi2, p*
**Education (%)**				48.67 *p* <0.000	25.40 *p* =0.000
None	40.0	26.6	33.3		
Primary	39.3	21.3	39.3		
Secondary	11.4	23.1	65.5		
Tertiary	10.5	26.3	63.2		
**Sex**				2.77 *p* = 0.251	0.74 *p* =0.389
Female	19.7	21.6	58.7		
Male	14.4	25.1	60.5		
**Residence**				5.50 *p* = 0.064	8.10 *p* =0.004
Urban	14.7	23.9	61.4		
Rural	23.5	21.3	55.2		
**Employment**				16.22 *p* <0.000	0.63 *p* =0.426
Not working	24.5	17.8	57.7		
Working	11.9	27.1	61.0		
**Age**				12.20 *p* =0.058	5.26 *p* =0.154
15–24	13.7	28.5	57.8		
25–34	12.2	21.4	66.4		
35–44	16.8	27.7	55.5		
45+	23.7	17.8	58.5		
**Source of Health Information**				10.78 *p* =0.005	9.69 *p* =0.002
No formal sources	37.5	16.7	45.8		
Uses formal sources	15.8	24.0	60.2		

Note: * Analysis based on the total score of literacy (0–16)

### Internal consistency and reliability

The corrected item-total correlation for items was high with a range from 0.53 to 0.78. The results obtained for the HL scale support its reliability, with a Cronbach’s alpha of *α* = 0.924 and the McDonald’s omega coefficient of ω = 0.929. Bartlett’s test of sphericity was significant x^2^(120) = 4609.123 (*p* < 0.001) with a Kaiser-Meyer-Olkin (KMO) value = 0.929 indicated that the data were suitable for factor analysis. Exploratory factor analysis using principal component extraction supported a single dominant factor with an eigenvalue of 7.7, explaining 49.0 per cent of the total variance. The second component had an eigenvalue of 1.5, explaining about 10.0% of variance, with all subsequent components having eigenvalues below 1. Factor loadings on the first component ranged from 0.50 to 0.79 (Table S1 in the supplementary materials), with all 16 items loading strongly and positively, supporting the unidimensional structure of the HLS-EU-Q16 in the Gabonese context. The scree plot showed a clear break after the first component, supporting retention of a single factor (Figure S2 in the supplementary materials).

### Behavioural differences across health literacy

Table 6 shows the distribution of malaria-related behaviours across HL. Significant associations were observed for dosing knowledge (χ²=17.1, *p* < 0.001), asking healthcare providers questions (χ²=24.47, *p* < 0.001), prompt treatment-seeking for self (χ²=16.38, *p* < 0.001), prompt treatment-seeking for family (χ²=13.71, *p* = 0.001), medication adherence (χ²=13.49, *p* = 0.001), and indoor spraying (χ²=23.13, *p* < 0.001). Among individuals with correct dosing knowledge, 66.0% had sufficient HL compared with 48.6% among those without dosing knowledge. Similarly, 65.4% of those who frequently asked healthcare providers questions had sufficient HL, compared with 41.5% among those who rarely or never did so. Regular bednet use (χ²=1.95, *p* = 0.376) and bush clearing (χ²=1.02, *p* = 0.600) showed no statistically significant association with HL categories. The Kruskal-Wallis tests confirmed these patterns using the continuous HL score, with dosing knowledge (H = 21.89, *p* < 0.001) and asking questions (H = 29.12, *p* < 0.001) showing the strongest gradients. Table 7 reveals similar patterns when the analysis focuses on Likert-scale assessment of the behaviours. Asking questions (*p* < 0.001) and medication adherence (*p* = 0.032) were significantly related to HL.

**Table 6:** Variations in binary health behaviours by health literacy

	HL Categories				HL score
Malaria Behaviour	Inadequate HL	Problematic HL	Sufficient HL	Chi^2^ p-value^a^	k-wallis^b^ (HL score)
Regular Bednet use				1.95 p= 0.376	1.05 p=0.306
Yes	17.69	21.09	61.22		
No	16.67	26.47	56.86		
Prompt treatment-seeking (self)				16.38 p<0.001***	5.37 p=0.021**
Yes	20	17.85	62.15		
No	12.21	33.14	54.65		
Dosing knowledge				17.13 p<0.001***	21.89 p<0.001***
Yes	12.70	21.27	66.03		
No/Not sure	24.86	26.52	48.62		
Indoor spraying				23.13 p<0.001***	0.19 p=0.665
Yes	10.1	32.21	57.69		
No/Not sure	22.3	16.72	60.98		
Medication adherence				13.49 p=0.001**	9.71 p=0.002**
Yes	17.38	20.24	62.38		
No	16.88	38.96	44.16		
Bush Clearing				1.02 p=0.600	0.04 p=0.842
Yes	16.71	23.67	59.63		
No/Not sure	20.31	18.75	60.94		
Prompt treatment-seeking (family)				13.71 p= 0.001**	7.31 p=0.007**
Yes	18.64	18.34	63.02		
No	14.47	33.33	52.2		
Asking questions				24.47 p<0.001***	29.12 p<0.001***
Yes	13.62	20.98	65.40		
No	29.27	29.27	41.46		

Notes: ^a^Chi-square test examines association between categorical HL levels (inadequate/ problematic/ sufficient) and binary behaviours (yes/no). It tests whether the distribution of HL categories differs between those who do/don’t engage in the behaviour. ^b^Kruskal-Wallis test examines association between continuous HL score (0-16 points) and binary behaviours. Tests whether median HL scores differ between those who do/don’t engage in the behaviour. ***, **, * significant at the 1, 5, and 10%-level. HL categories: Inadequate (0-8 points), Problematic (9-12 points), Sufficient (13-16 points). Behaviours dichotomised as: Yes (always/often) vs. No (sometimes/rarely/never)

**Table 7:** Variations in Likert Scale health behaviours by health literacy

Malaria Behaviour	Chi^2^ *p*-value^a^	k-wallis^b^ (HL score)
Bednet use	*p* = 0.934	*p* = 0.773
Prompt treatment-seeking (self)	*p* = 0.310	*p* = 0.049**
Medication adherence	*p* = 0.032**	*p* = 0.052*
Prompt treatment-seeking (family)	*p* = 0.571	*p* = 0.219
Asking questions	*P* < 0.001***	*p* = 0.005**

Notes: Behaviours measured on 5-point Likert scale (0=never to 4=always). ^a^Chi-square tests association between categorical HL levels (inadequate/ problematic/ sufficient) and ordinal behaviour responses. HL levels computed as: inadequate (0 to 8), problematic (9 to 12), and sufficient (13 to 16). ^b^Kruskal-Wallis tests association between continuous HL score (0-16) and ordinal behavior responses. ***, **, * significant at 1%, 5%, and 10% levels

### Associations between health literacy and malaria control behaviours

Tables 8 and 9 present the average marginal effects after binary logistic regressions and ordered logistic regression results from the multivariate models. Higher HL was significantly associated with a greater likelihood of dosing knowledge, asking healthcare providers questions, and medication adherence. In the fully adjusted model (Model c), a one-point increase in the HL scale was associated with a 2.2% point increase in the probability of correct dosing knowledge (AME = 0.022, 95% CI: 0.013–0.032, *p* < 0.001), a 1.8% point increase in the probability of asking healthcare providers questions (AME = 0.018, 95% CI: 0.009–0.027, *p* < 0.001), and a 1.3% point increase in the probability of medication adherence (AME = 0.013, 95% CI: 0.005–0.022, *p* < 0.01). The ordered logistic regression results show similar findings: a one-point increase in HL increased the odds of moving to a higher category of asking questions by approximately 9% (OR = 1.092, 95% CI: 1.046–1.141, *p* < 0.001) and of medication adherence by approximately 9% (OR = 1.089, 95% CI: 1.043–1.137, *p* < 0.001). Regular bednet use showed a notable pattern: the association of regular bednet use with HL was non-significant in the unadjusted model (AME = 0.001, 95% CI: -0.007-0.010) but became statistically significant following adjustment for socioeconomic and contextual covariates (AME = 0.012, 95% CI: 0.001–0.023, *p* < 0.05), a finding discussed further below. Prompt treatment-seeking (self and family), indoor spraying, and bush clearing were not significantly associated with HL in the multivariate models.

**Table 8:** Average marginal effects of health literacy on binary malaria health behaviours

Malaria Behaviour	Health literacy		
	AME^a^ (95% CI)	AME^b^ (95% CI)	AME^c^ (95% CI)
Regular bednet use	0.001 [-0.007,0.010]	0.011** [0.001,0.022]	0.012** [0.001,0.023]
Prompt treatment-seeking (self)	0.001 [-0.007,0.009]	0.005 [-0.005,0.015]	0.004 [-0.006,0.015]
Dosing knowledge	0.015*** [0.007,0.023]	0.023*** [0.013,0.032]	0.022*** [0.013,0.032]
Indoor spraying	0.005 [-0.003,0.013]	0.006 [-0.004,0.015]	0.006 [-0.004,0.015]
Medication adherence	0.008* [0.001,0.014]	0.013** [0.005,0.020]	0.013** [0.005,0.022]
Bush Clearing	0.004 [-0.001,0.011]	0.006 [-0.002,0.014]	0.004 [-0.004,0.011]
Prompt treatment-seeking (Family)	0.001 [-0.007,0.009]	0.007 [-0.003,0.017]	0.007 [-0.003,0.017]
Asking questions	0.016*** [0.010,0.023]	0.019*** [0.011,0.028]	0.018*** [0.009,0.027]

Notes: ***, **, * significant at the 1, 5, and 10%-level, respectively. ^a^unadjusted coefficients. ^b^Coefficients adjusted for age, sex, residence, employment, recent malaria exposure, insurance status, proximity to health centres, source of health information, and availability of community health resources (including malaria prevention programs). ^c^additionally adjusts for education level. All models estimated using robust standard errors. CI: confidence interval. AME = Average Marginal Effects and represent changes in predicted probability of the behaviours per one-unit increase in HL. Analysis based on binary measures of the malaria behaviours. For behaviours originally measured on five-point Likert scales (bednet use, treatment-seeking, medication adherence, asking questions), responses were dichotomized as consistent (Always/Often) vs. inconsistent (Sometimes/Rarely/Never). See Table 9 for ordered logistic regression results preserving the full ordinal structure

**Fig. 1:**
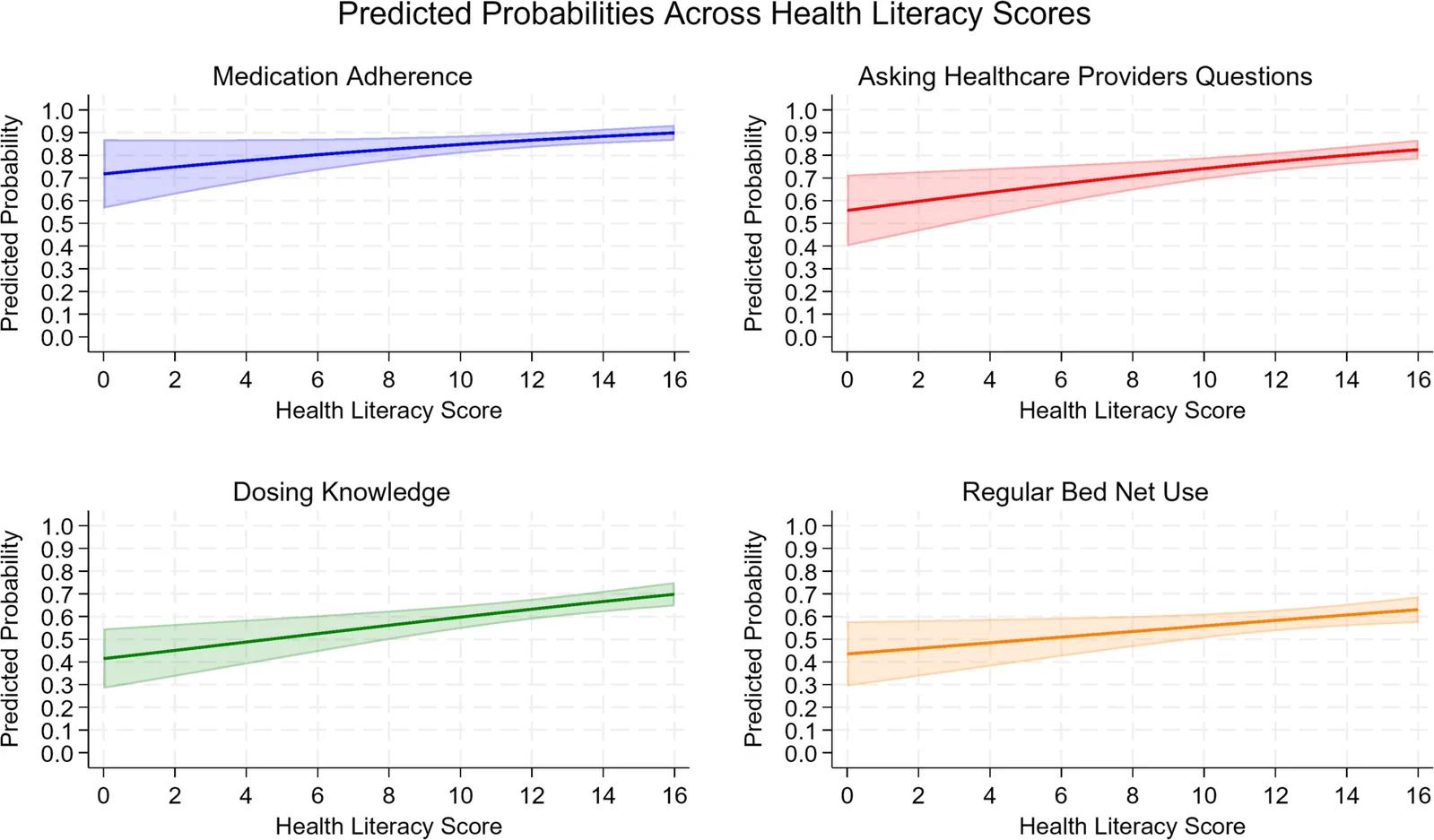
Predicted probabilities of malaria-related behaviours across health literacy scores

Figure 1 presents the predicted probabilities of four malaria-related behaviours across the full range of HL scores (0 to 16), with all covariates held at their observed means (Model c). Across all four panels, a consistent positive gradient is evident, indicating that higher HL scores are associated with greater predicted probabilities of each behaviour. The steepest gradients are observed for asking healthcare providers questions and dosing knowledge, where predicted probabilities rise noticeably across the score range reflecting the largest average marginal effects among the four outcomes (AME = 0.018 and AME = 0.022). The gradient for medication adherence, though positive, is comparatively modest. The widening confidence intervals at the lower end of the score distribution across all panels reflect the left-skewed distribution of HL scores in the sample (Figure S1 in the supplementary materials); findings in this range should therefore be interpreted with caution.

**Table 9:** Association and effects of health literacy on malaria health behaviours

Malaria Behaviour	Health literacy		
	Unadjusted OR (95% CI)	Adjusted OR^a^ (95% CI)	Adjusted OR^b^ (95% CI)
Bednet use	1.002 [0.971,1.037]	1.032 [0.992,1.074]	1.034 [0.993,1.076]
Prompt treatment-seeking (self)	1.006 [0.976,1.038]	1.011 [0.977,1.048]	1.012 [0.975,1.052]
Medication adherence	1.067*** [1.028,1.106]	1.083*** [1.039,1.129]	1.089*** [1.043,1.137]
Prompt treatment-seeking (family)	1.017 [0.985,1.052]	1.026 [0.988,1.065]	1.031 [0.992,1.072]
Asking questions	1.119*** [1.072,1.167]	1.101*** [1.054,1.151]	1.092*** [1.046,1.141]

Notes: ***, **, * significant at the 1, 5, and 10%-level, respectively. ^a^ Coefficients adjusted for age, sex, residence, employment, recent malaria exposure, insurance status, proximity to health centres, source of health information, and availability of community health resources (including malaria prevention programs). ^b^additionally adjusts for education level. All models estimated using robust standard errors. OR= Odds Ratios, CI = Confidence interval. Analysis based on Likert scale measurement (Ordered Logistic Model). Analysis based on Likert scale measurement (Ordered Logistic Model) preserving the full ordinal structure (Always/Often /Sometimes/ Rarely/ Never). Only behaviours originally measured on five-point scales are included

## Discussion

The study evaluated the psychometric performance of the French HLS-EU-Q16 within a Gabonese sample and examined associations between HL and malaria-related behaviours. The scale demonstrated adequate psychometric properties, including acceptable internal consistency, a unidimensional factor structure, a coherent Rasch difficulty hierarchy, and construct validity through known-groups discrimination and behavioural associations. Item-level response patterns revealed differential difficulty across HL domains, with healthcare interaction tasks like following medical instructions considerably easier than health promotion tasks like accessing mental health information, indicating domain-specific literacy competencies rather than uniform literacy levels. The levels of sufficient HL (59.7%) are comparable to a study from Switzerland [56] where sufficient levels of HL were reported. However, they differ from previous studies, in which a high percentage of the sample had insufficient levels of HL [57].

HL varied systematically by education levels. The results show that individuals with no formal education and primary education had significantly lower levels of HL compared to those with secondary and tertiary education. No statistically significant differences in HL levels were by age. This finding contrasts with the results in Europe, where older people had insufficient literacy levels [32]. Exploratory factor analysis of the 16 items of the French HLS-EU-Q16 items determined a single factor structure accounting for 49 per cent of the total variance, consistent with validations in Sweden using the Arabic version of the instrument [48] as well as Swedish parents with infant children [58]. However, the results differ from those found in Bangladesh [59], Romania [60], where three- and four-factor structures were found, respectively. This demonstrates that the HL dimensions of the scale manifest themselves differently across cultures. Mokken analysis confirmed scale homogeneity, with Loevinger’s H coefficients ranging from 0.42 to 0.73 (all greater than the 0.30 threshold), indicating items measure a single underlying construct. Item difficulty parameters revealed substantial heterogeneity, ranging from − 1.98 to + 2.48 logits, indicating the scale covers a broad difficulty spectrum. The global item-trait interaction test and the Andersen likelihood ratio test were statistically significant, and this behaviour is a recognised feature of fitting latent trait models in samples where the latent trait is highly skewed; it is consistent with prior validations of the HLS-EU-Q16, in which imperfect global fit has coexisted with acceptable item-level properties and a coherent difficulty structure [30]. The convergence of a theoretically meaningful difficulty hierarchy, acceptable item-level fit for the majority of items, and adequate scalability (reported via the Mokken analysis) provides reasonable assurance that the instrument functions as a valid measure of HL in this sample, notwithstanding the constraints on the global fit statistics.

The results show that people with higher HL scores showed better behaviours of malaria. Medication adherence and dosing knowledge showed significant positive associations with HL. This finding is consistent with literature demonstrating that individuals with higher HL better understand medication instructions, recognise the importance of completing prescribed courses, and are more likely to adhere to treatment regimens [61–63]. However, it contradicts findings that found no association between literacy levels and medication adherence [64]. Engagements and question-asking behaviour during healthcare encounters showed significant positive associations. The results suggest that HL is associated with how individuals engage with healthcare providers. Higher HL appears to empower patients to actively participate in their care through information-seeking behaviours, which can lead to improved understanding of conditions and treatments. It should be noted, however, that the propensity to ask questions of providers is not a pure measure of HL and is likely also influenced by prior health system experiences, interpersonal confidence, and perceived power dynamics in the patient-provider relationship, factors that may operate independently of HL [3].

HL enables individuals to better understand and implement preventive health measures [65]. The relationship between HL and bednet use revealed a statistically significant positive relationship when other factors are controlled for, consistent with a possible suppression effect [66] in the model. Residence for example, may contribute to this pattern: rural respondents in our sample exhibit both lower HL and higher bed net use. Higher bed net use in rural areas could be driven by greater malaria exposure and the reach of community-based distribution programmes [67]. Therefore, once residence and related contextual covariates are controlled for, the positive relationship between HL and bed net use becomes evident. However, this interpretation remains speculative in cross-sectional data and cannot be confirmed from the present analysis. This adjusted association should be interpreted as a conditional relationship after accounting for structural and contextual factors rather than as evidence of a direct behavioural effect of HL on bednet use. There exist no statistically significant association between personal treatment-seeking behaviour and family treatment-seeking behaviour and IRS. This absence of association may reflect several underlying factors. Treatment-seeking behaviour may be more strongly associated with structural barriers such as healthcare accessibility, affordability, and availability rather than individual knowledge levels [68–70]. Cultural beliefs and traditional healing practices may override HL considerations when making immediate treatment decisions [18, 71, 72]. Similarly, the uptake of IRS is frequently shaped by community-level interventions, government campaigns, availability of spraying services, and trust in public health programs rather than individual knowledge alone [73, 74].

To ensure the validity of our findings, we tested the robustness of our results using alternative model specifications. The results, presented in Table S6 in the Supplementary materials, confirm that our main conclusions hold across different estimation frameworks and measurement approaches. First, we employed a mediation framework to test whether dosing knowledge and asking questions mediated the relationships between HL and health behaviours. Contemporary mediation analysis [75, 76] recognises that mediation can occur even in the absence of a significant total effect, representing patterns in which HL associates with the behaviours entirely through the mediator (indirect-only mediation). Therefore, variables which showed no direct association with HL were included in mediation analyses to test whether HL is associated with these behaviours through mediators. We estimated our models by utilising dosing knowledge and asking questions as mediators in the relationships between HL and malaria-control behaviours.

The mediation analysis (Models 1 and 2) examined whether the relationship between HL and malaria behaviours operated through: asking healthcare providers questions (Model 1) and dosing knowledge (Model 2). For treatment-seeking behaviours, the association between HL and this outcome was no longer evident once asking questions was included, a pattern consistent with mediation through this enabling variable. For both self-treatment-seeking and family treatment-seeking, the direct effects became non-significant once asking questions was included in the model, a pattern that would be consistent with HL relating to treatment-seeking via patient-provider communication, though the cross-sectional design cannot establish this directionality. A similar pattern was observed for bed net use in relation to asking questions in Model 1. For treatment adherence, HL showed partial mediation through both asking questions and dosing knowledge. Indoor spraying and bush clearing showed neither significant total effects nor mediation, reinforcing earlier findings that these behaviours could be largely determined by structural and community-level factors rather than individual HL. Including community fixed effects based on the areas of data collection (Model 3) to control for unobserved spatial heterogeneity did not alter these patterns, confirming that the observed associations reflect individual-level HL effects rather than unmeasured community characteristics. These results are consistent with HL relating to malaria prevention behaviours via communicative and knowledge-based pathways, although these mediation results should be regarded as exploratory and hypothesis-generating rather than confirmatory given the cross-sectional design.

Our study had some limitations. First, sampling challenges affected generalisability. Although part of our sample used systematic probability sampling with complete household registries, Lambaréné required stratified convenience sampling with neighbourhood quotas. This approach may limit generalisability. Second, the cross-sectional design precludes causal inference. Observed associations between HL and behaviour could reflect bidirectional relationships. For the same reason, the mediation results should be read as exploratory and hypothesis-generating; they describe statistical patterns consistent with mediation but cannot demonstrate that HL operates causally through the proposed pathways. Third, self-reported behaviours may be subject to social desirability bias. Self-reported bednet use, medication adherence, and treatment-seeking are especially susceptible to this form of response bias in community health surveys, where respondents may perceive a socially normative answer; the likely direction of this bias is upward, favouring over-reporting of health-protective behaviours, which may inflate the observed associations. We used private interviews with trained interviewers to minimise this concern. Fourth, several behavioural outcomes are conceptually broad rather than malaria-specific: the treatment-seeking items capture care-seeking for symptoms suggestive of malaria (fever or cough) rather than for parasitologically confirmed malaria, and medication adherence was assessed as completion of any prescribed course, whether antimalarial or other treatment. This broader framing may attenuate the specificity of the observed associations, and future research employing point-of-care diagnostic confirmation and treatment-specific adherence measures would help isolate malaria-specific behaviours more precisely. Dichotomising Likert-scale outcomes reduces statistical power but enhances interpretability. Importantly, consistency of findings across ordered and binary logistic regression approaches strengthens confidence in observed associations. Unmeasured confounding remains possible. Despite controlling for demographic and health system factors, we did not measure trust in healthcare, recourse to traditional medicine, household decision-making dynamics, household wealth, or culturally specific health beliefs, factors that may be associated with both HL and behaviours. The ceiling effect observed with the dichotomised scale (25.6% at maximum score) limits precision for individuals with high HL. This measurement limitation is inherent to the HLS-EU-Q16 analytical framework rather than specific to our study design [30]. For the current study’s objective of examining HL’s role in malaria prevention behaviours, the dichotomised scale was adequate as the scale demonstrated strong psychometric properties (reliability, validity, known-groups discrimination and Rasch analysis) and support its utility for assessing HL within our sample. However, from the original scale, only 0.4% of participants scored at maximum on the four-point Likert scale, suggesting future research requiring discrimination across the full HL spectrum should consider retaining the original response format. We therefore recommend that future research validate this scale in larger and more balanced samples that achieve fuller representation across the entire HL continuum, particularly at the lower end of the scale, and that complementary methodological approaches such as confirmatory factor analysis be applied to triangulate the structural validity evidence reported here. The three items exhibiting significant misfit (items 1, 14, and 15) may also warrant closer examination of their translation and cultural adaptation in future applications of the instrument.

Despite these limitations, the study strengths include adequate statistical power from a large sample size, use of an internationally validated and locally adapted HL instrument, geographic diversity across urban-rural areas, and consistency with theoretical predictions and evidence from comparable sub-Saharan African contexts. The findings of our study apply to adult populations from comparable malaria-endemic regions and with socioeconomic and health system profiles comparable to the study sites; caution is warranted in extrapolating to regions with different malaria transmission dynamics, healthcare access, or net distribution infrastructure. Future research should use longitudinal designs and examine additional health outcomes and behaviours, including vaccination behaviours.

## Conclusion

This study makes two important contributions. First, it provides evidence of acceptable psychometric performance for a culturally adapted HL instrument within this Gabonese sample. The HLS-EU-Q16 scale demonstrated adequate internal consistency and successfully discriminated between population subgroups based on education level and rural-urban residence. These findings reflect performance within the present sample, and broader validation across the wider Gabonese population will require confirmation in larger, probability-based samples. This validated instrument can support future HL assessments and enable evaluation of health promotion programmes in similar areas. Second, for malaria control efforts, this study provides empirical evidence linking HL to specific malaria prevention and treatment behaviours. After adjusting for demographic, socioeconomic, and health environment factors, HL demonstrated significant independent associations with four behaviours: dosing knowledge, patient-provider communication through asking questions, medication adherence, and bed net use. Notably, the bed net association only emerged after controlling for covariates, indicating that adjustment for covariates revealed HL’s independent contribution to bed net use. In contrast, treatment-seeking, IRS and bush clearing showed no independent associations with HL, suggesting these may be driven primarily by structural factors and community-level interventions. The mediation patterns observed are consistent with HL relating to behaviour primarily via cognitive and communicative pathways, particularly patient-provider communication and dosing knowledge, though this interpretation is exploratory. For malaria control, these findings indicate that literacy-enhancing interventions should focus on medication education and healthcare communication skills, while recognizing that structural interventions remain essential for addressing treatment access barriers.

## Supplementary Information

Below is the link to the electronic supplementary material.

Supplementary Material 1

Supplementary Material 2

## Data Availability

The data analysed during the study is available from the corresponding author on reasonable request.

## Abbreviations

HL: Health Literacy
IRS: Indoor residual spraying
