# Supplementary material for: Linking health literacy to malaria prevention and treatment behaviours: evidence from a community-based study in Gabon
Source: BMC Health Serv Res. 2026 Aug 24;26:1178. doi: 10.1186/s12913-026-15138-1 (PMC13508316; doi:10.1186/s12913-026-15138-1)
Supplement: Supplementary file 1 [file 12913_2026_15138_MOESM1_ESM.docx]

**Supplementary materials**

**Figure S1: Distribution of Health Literacy**

**Figure S2: Scree plot after factor analysis**

**Table S1: Factor loadings**

| **Item** | **Factor 1** | **Factor 2** |
| --- | --- | --- |
| 1 | 0.59 | -0.33 |
| 2 | 0.66 | -0.38 |
| 3 | 0.75 | -0.42 |
| 4 | 0.79 | -0.33 |
| 5 | 0.69 | -0.27 |
| 6 | 0.75 | -0.24 |
| 7 | 0.75 | -0.29 |
| 8 | 0.50 | 0.28 |
| 9 | 0.68 | 0.11 |
| 10 | 0.60 | 0.40 |
| 11 | 0.71 | 0.34 |
| 12 | 0.76 | 0.30 |
| 13 | 0.73 | 0.28 |
| 14 | 0.64 | 0.30 |
| 15 | 0.78 | 0.18 |
| 16 | 0.69 | 0.20 |
| Eigen value | 7.77 | 1.46 |
| Variance Proportion | 0.49 | 0.09 |

**Rasch Model Diagnostics and Differential Item Functioning using Joint Maximum likelihood**

A joint maximum likelihood (JML) Rasch model in R was used to estimate separation reliability indices, person and item separation reliability and differential item functioning. Item fit statistics were estimated with joint maximum likelihood (JML) estimation and DIF was assessed using logistic regression methods. Person separation reliability = 0.745; item separation reliability = 0.984.

**Table S2: Item fit statistics from Rasch analysis (JML estimation)**

| **HL Item** | **p-value** | **Outfit MSQ** | **Infit MSQ** | **Outfit t** | **Infit t** | **Discrim.** |
| --- | --- | --- | --- | --- | --- | --- |
| 1 | **<0.001** | **1.523** | 1.225 | **2.646** | 2.488 | 0.339 |
| 2 | 0.099 | 1.094 | 0.992 | 0.614 | -0.072 | 0.515 |
| 3 | 0.893 | 0.906 | 1.022 | -0.333 | 0.241 | 0.496 |
| 4 | 1.000 | 0.612 | 0.755 | -1.057 | -1.764 | 0.569 |
| 5 | 0.209 | 1.057 | 1.055 | 0.491 | 0.825 | 0.488 |
| 6 | 1.000 | 0.731 | 0.967 | -1.100 | -0.266 | 0.514 |
| 7 | 1.000 | 0.525 | 0.643 | -1.350 | **-2.686** | 0.600 |
| 8 | 0.059 | 1.116 | 1.086 | 0.624 | 1.436 | 0.233 |
| 9 | 0.996 | 0.812 | 0.947 | -1.339 | -0.690 | 0.542 |
| 10 | 0.425 | 1.009 | 1.067 | 0.115 | 1.210 | 0.366 |
| 11 | 0.988 | 0.837 | 0.863 | -1.494 | -2.468 | 0.541 |
| 12 | 0.999 | 0.788 | 0.869 | -1.990 | -2.208 | 0.580 |
| 13 | 1.000 | 0.714 | 0.843 | -2.069 | -2.120 | 0.606 |
| 14 | **0.002** | 1.224 | 1.124 | 1.231 | 1.400 | 0.413 |
| 15 | 1.000 | 0.654 | 0.798 | **-2.708** | **-2.880** | 0.636 |
| 16 | 0.996 | 0.809 | 0.947 | -1.398 | -0.704 | 0.538 |

*Note. Outfit and Infit MSQ values outside the conventional 0.5–1.5 range are shown in bold. Outfit t and Infit t values exceeding ±2.5 are shown in bold. Discrim. = item discrimination. Items 1 and 14 show significant chi-square fit (p < 0.05); item 1 additionally exceeds the MSQ upper bound. The item-fit pattern corroborates the conditional maximum likelihood (CML) estimates in Table 4 of the main manuscript; difficulty parameters differ modestly between the two tables because JML and CML use different estimation routines, while the rank ordering of item difficulties is preserved.*

**Table S3: Differential Item Functioning by sex (reference group: male)**

| **HL Item** | **LRT Statistic** | **p-value** | **Nagelkerke R²** | **ZT** | **JG** | **DIF** |
| --- | --- | --- | --- | --- | --- | --- |
| 1 | 0.86 | 0.64 | 0.002 | A | A |  |
| 2 | **8.41** | **0.01** | 0.017 | A | A | ***** |
| 3 | 0.84 | 0.65 | 0.002 | A | A |  |
| 4 | 0.46 | 0.79 | 0.001 | A | A |  |
| 5 | 1.60 | 0.44 | 0.003 | A | A |  |
| 6 | 3.19 | 0.20 | 0.008 | A | A |  |
| 7 | 0.79 | 0.67 | 0.002 | A | A |  |
| 8 | 2.63 | 0.26 | 0.004 | A | A |  |
| 9 | 1.77 | 0.41 | 0.003 | A | A |  |
| 10 | 0.05 | 0.97 | 0.000 | A | A |  |
| 11 | 1.75 | 0.41 | 0.002 | A | A |  |
| 12 | 1.15 | 0.56 | 0.001 | A | A |  |
| 13 | **6.74** | **0.03** | 0.012 | A | A | ***** |
| 14 | 1.71 | 0.42 | 0.004 | A | A |  |
| 15 | 0.37 | 0.82 | 0.001 | A | A |  |
| 16 | **6.81** | **0.03** | 0.013 | A | A | ***** |

*Note. DIF assessed using logistic regression. Detection threshold: LRT statistic > 5.991 (χ² with df=2, p=0.05). Items flagged (*) showed borderline statistical significance but all effect sizes were negligible by both Zumbo-Thomas (ZT) and Jodoin-Gierl (JG) criteria (all R² < 0.02). No item survives Bonferroni correction (adjusted α = 0.003). ZT effect size codes: A = negligible (R² < 0.13), B = moderate, C = large. JG effect size codes: A = negligible (R² < 0.035), B = moderate (≥0.035), C = large (≥0.07).*

**Table S4: Differential Item Functioning by age group (reference group: 15–24 years)**

| **HL Item** | **LRT Statistic** | **p-value** | **Nagelkerke R²** | **ZT** | **JG** | **DIF** |
| --- | --- | --- | --- | --- | --- | --- |
| 1 | 6.90 | 0.54 | 0.016 | A | A |  |
| 2 | 12.32 | 0.13 | 0.025 | A | A |  |
| 3 | 9.02 | 0.33 | 0.023 | A | A |  |
| 4 | **19.13** | **0.01** | **0.058** | A | **B** | ***** |
| 5 | 10.60 | 0.22 | 0.019 | A | A |  |
| 6 | 7.88 | 0.44 | 0.020 | A | A |  |
| 7 | **26.91** | **0.00** | **0.079** | A | **C** | ***** |
| 8 | 8.99 | 0.34 | 0.014 | A | A |  |
| 9 | **16.70** | **0.03** | 0.031 | A | A | ***** |
| 10 | **16.55** | **0.03** | 0.026 | A | A | ***** |
| 11 | 5.02 | 0.75 | 0.007 | A | A |  |
| 12 | **15.91** | **0.04** | 0.024 | A | A | ***** |
| 13 | 11.71 | 0.16 | 0.021 | A | A |  |
| 14 | 9.38 | 0.31 | 0.021 | A | A |  |
| 15 | 12.98 | 0.11 | 0.022 | A | A |  |
| 16 | 13.22 | 0.10 | 0.024 | A | A |  |

*Note. DIF assessed using logistic regression with 4 focal groups (25–34, 35–44, 45–54, 55+). Detection threshold: LRT statistic > 15.507 (χ² with df=8, p=0.05). Bold R² values indicate moderate or large effect size by Jodoin-Gierl criteria (R² ≥ 0.035). Items 4 and 7 show meaningful DIF by effect size (JG grade B and C respectively); remaining flagged items show negligible effect.*

**Table S5: Differential Item Functioning by education level (reference group: secondary education)**

| **HL Item** | **LRT Statistic** | **p-value** | **Nagelkerke R²** | **ZT** | **JG** | **DIF** |
| --- | --- | --- | --- | --- | --- | --- |
| 1 | 5.16 | 0.52 | 0.012 | A | A |  |
| 2 | 4.09 | 0.66 | 0.008 | A | A |  |
| 3 | 10.56 | 0.18 | 0.026 | A | A |  |
| 4 | **14.25** | **0.02** | **0.043** | A | **B** | ***** |
| 5 | **12.80** | **0.04** | 0.024 | A | A | ***** |
| 6 | 2.61 | 0.85 | 0.006 | A | A |  |
| 7 | **20.36** | **0.00** | **0.060** | A | **B** | ***** |
| 8 | 5.39 | 0.49 | 0.008 | A | A |  |
| 9 | 10.85 | 0.09 | 0.020 | A | A |  |
| 10 | **21.14** | **0.00** | 0.033 | A | A | ***** |
| 11 | 12.29 | 0.05 | 0.017 | A | A |  |
| 12 | 9.27 | 0.15 | 0.014 | A | A |  |
| 13 | 2.81 | 0.83 | 0.005 | A | A |  |
| 14 | **13.90** | **0.03** | 0.031 | A | A | ***** |
| 15 | **15.26** | **0.01** | 0.025 | A | A | ***** |
| 16 | 7.04 | 0.31 | 0.013 | A | A |  |

*Note. DIF assessed using logistic regression with 3 focal groups (none, primary, tertiary). Secondary education used as reference group (n=342) as the largest subgroup. Detection threshold: LRT statistic > 12.592 (χ² with df=6, p=0.05). Bold R² values indicate moderate effect size by Jodoin-Gierl criteria (R² ≥ 0.035). Items 4 and 7 show moderate DIF (JG grade B); all other flagged items show negligible effect sizes. Estimates for the no-education subgroup (n=15) should be interpreted with caution given the very small sample.*

**Table S6: Mediation Analysis**

| **Malaria Behaviour** | **Model 1 [95% CI]** | | | **Model 2 [95% CI]** | | | **Model 3** |
| --- | --- | --- | --- | --- | --- | --- | --- |
|  | **TE** | **DE** | **IE** | **TE** | **DE** | **IE** | **AME [95% CI]** |
| Prompt treatment-seeking (self) | 0.14**  [0.03, 0.25] | 0.001  [-0.06, 0.06] | 0.14***  [0.05, 0.23] | 0.11**  [0.02, 0.20] | 0.0004  [-0.06, 0.06] | 0.11***  [0.04, 0.18] | 0.004  [-0.01,0.02] |
| Dosing Knowledge | 0.16***  [0.08, 0.23] | 0.10***  [0.05, 0.15] | 0.06*  [-0.004, 0.12] |  | | | 0.02***  [0.01,0.03] |
| Treatment Adherence | 0.30***  [0.15, 0.44] | 0.08**  [0.01, 0.15] | 0.22***  [0.09, 0.35] | 0.18***  [0.10, 0.27] | 0.09***  [0.03, 0.16] | 0.09**  [0.02, 0.17] | 0.01***  [0.01,0.02] |
| Prompt treatment-seeking (family) | 0.17**  [0.05, 0.27] | 0.02  [-0.04, 0.07] | 0.15***  [0.06, 0.24] | 0.10***  [0.03, 0.18] | 0.03  [-0.03, 0.09] | 0.07**  [0.02, 0.13] | 0.01  [-0.003,0.02] |
| Indoor Spraying | 0.06  [-0.02, 0.13] | 0.03  [-0.03, 0.08] | 0.03  [-0.03, 0.09] | 0.06*  [-0.00,-0.13] | 0.03  [-0.02,0.08] | 0.03  [-0.02, 0.09] | 0.01  [-0.004,0.02] |
| Bush clearing | 0.08  [-0.02, 0.18] | 0.04  [-0.03, 0.12] | 0.04  [-0.04, 0.11] | 0.08*  [-0.004,0.17] | 0.05  [-0.03, 0.13] | 0.03  [-0.03, 0.10] |  |
| Bednet use | 0.13***  [0.05, 0.21] | 0.04  [-0.01, 0.09] | 0.09**  [0.02, 0.16] | 0.06**  [0.01, 0.12] | 0.05**  [0.002,0.10] | 0.01  [-0.03, 0.06] |  |
| Asking questions |  | | | 0.16***  [0.09, 0.24] | 0.11***  [0.05, 0.17] | 0.05*  [-0.00, 0.11] | 0.02***  [0.01,0.03] |

*Notes: Estimates are presented as coefficients (95% confidence interval) unless otherwise indicated. ***, **, * significant at the 1, 5, and 10%-level respectively. CI = Confidence interval; AME = Average Marginal Effects. Model 1 is the mediation model using asking questions. Model 2 is the mediation model using dosage knowledge. All models were adjusted for age, sex, residence, employment, recent malaria exposure, insurance status, proximity to health centres, availability of community health resources, and source of health information, and estimated using robust standard errors. TE Total effect estimates, DE Direct effect, IE Indirect effect. Model 3 includes community fixed effects.*
