# Supplementary material for: Linking health literacy to malaria prevention and treatment behaviours: evidence from a community-based study in Gabon
Source: BMC Health Serv Res. 2026 Aug 24;26:1178. doi: 10.1186/s12913-026-15138-1 (PMC13508316; doi:10.1186/s12913-026-15138-1)
Supplement: Supplementary file 2 [file 12913_2026_15138_MOESM2_ESM.docx]

**Questionnaire**

Record ID _______________

Site: Lambaréné ☐ Four-place ☐ Sindara ☐ Other ☐

Date: __/__/____

Is the household part of demographic surveillance Yes ☐ No ☐

If yes, Household number________________________

**Section A: Demographic Information**

A1. Do you know your date of birth? Yes☐ No ☐

A2: If Yes in A1, Date of birth __/__/____ ; If No, please state Age

A3. Sex: Male☐ Female ☐

A4. Education Level: None☐ Primary ☐ Secondary ☐ Tertiary ☐

A5. Marital Status: Single ☐ Married ☐ Divorced ☐ Widowed ☐ Cohabitation ☐

A6. Number of people currently living in your household including you: _______

A6. Number of people

a) Under the age of 5 _____
b) Above the age of 5 _____

A7. Area of residence Rural ☐ Urban ☐

**Section B: Economic Factors**

B1. Employment Status: Employed ☐ Unemployed ☐ Self-employed ☐ Retired ☐

B2. Occupation: ___________________

B3. Health Insurance Status: Insured ☐ Uninsured ☐

B4. If Insured, covered by:  Public health insurance (CNAMGS) ☐ Private Health Insurance ☐ Others ☐ Specify____________________________

**Section C: Health Environment**

C1. Availability of Health Services (e.g., clinics, hospitals) in your area: Yes ☐ No ☐

C2. Closeness of the nearest health facility to your residence

Less than 1km ☐ 1km to 3kms ☐ 3kms to 5kms ☐ More than 5kms☐

C3. Community Health Resources (e.g., malaria prevention programs): Available ☐ Unavailable ☐

**Section D: Malaria Knowledge**

D1. Have you or any member of your family had a malaria experience in the past 2 weeks?

Yes ☐ No ☐ Not sure ☐

D2. Malaria is primarily transmitted by

Mosquito bites ☐ Contaminated water ☐ Person-to-person contact ☐

Other (please specify): ________________ No Idea ☐

D3. The preventive measures for avoiding malaria are (Select all that apply)

Sleeping under bed nets ☐ Using insect repellent/insecticide ☐ Taking anti-malarial medication ☐ Eliminating mosquito breeding sites ☐ Other (please specify): ____________________

D4. What are the most common symptoms of malaria? please select all that apply.

Fever ☐ Headaches ☐ Muscle pain ☐ Fatigue ☐ Lack of appetite ☐ Diarrhea ☐ Other _____________________________

D5. Information about malaria and its prevention/treatment is usually sourced from (Select all that apply)

Health centres/clinics ☐ Media (TV, radio, Phone) ☐ Community health workers ☐

Friends and family ☐ Other (please specify): _____________________________

**Section E: Health Literacy**

**Indicate, on a scale from very easy to very difficult, how easy it is for you to...**

| **Health Literacy** | **very easy** | **easy** | **difficult** | **very difficult** |
| --- | --- | --- | --- | --- |
| ... find information about malaria/disease treatments that affect you? | ☐ | ☐ | ☐ | ☐ |
| … know where to get professional help when you are sick? (e.g. doctor, nurse, pharmacist or psychologist) | ☐ | ☐ | ☐ | ☐ |
| … understand what a doctor is telling you? | ☐ | ☐ | ☐ | ☐ |
| … understand your doctor's or pharmacist's instructions on how to take your medication? | ☐ | ☐ | ☐ | ☐ |
| … know when it would be useful to have the opinion of another doctor? | ☐ | ☐ | ☐ | ☐ |
| … use the information the doctor gives you to make decisions about your disease or illness? | ☐ | ☐ | ☐ | ☐ |
| … follow the instructions of your doctor or pharmacist? | ☐ | ☐ | ☐ | ☐ |
| … find information on what to do in case of psychological problems? (e.g. stress, depression or anxiety) | ☐ | ☐ | ☐ | ☐ |
| … understand the warnings about the health impact of certain behaviours such as smoking, not exercising enough, and drinking too much? | ☐ | ☐ | ☐ | ☐ |
| … understand the information on recommended screenings and examinations? (e.g., cancer screening, blood sugar test) | ☐ | ☐ | ☐ | ☐ |
| … assess the reliability of information available in the media about what is dangerous to health? (e.g. newspapers, television or internet) | ☐ | ☐ | ☐ | ☐ |
| … know how to protect yourself from diseases from the information available in the media? (e.g. newspapers, television or internet) | ☐ | ☐ | ☐ | ☐ |
| … find out about activities that are beneficial to your health and well-being? (e.g. exercise, football, running) | ☐ | ☐ | ☐ | ☐ |
| … understand the health advice of your family or friends? | ☐ | ☐ | ☐ | ☐ |
| … understand the information available in the media to be healthier? | ☐ | ☐ | ☐ | ☐ |
| … identify what behaviors in your everyday life have an impact on your health? | ☐ | ☐ | ☐ | ☐ |

**Section F: Malaria Control Behaviours**

F1. How frequently do you use bed nets as a preventive measure against malaria?

Every night ☐ Most nights ☐ Occasionally ☐ Rarely ☐ Never ☐

F2. Do you participate in or is your house sprayed with indoor residual spraying for malaria control?

Yes ☐ No ☐ Not sure ☐

F3. Do you participate in clearing any bushes around your home?

Yes ☐ No ☐ Not sure ☐

F4. When facing any illness (fever/cough), I go to the health centre or speak with a health practitioner immediately.

Always ☐ Often ☐ Occasionally ☐ Rarely ☐ Never ☐

F5. If anyone in my household has a fever or cough, I immediately take them to see a doctor or tell them to see a doctor.

Always ☐ Often ☐ Occasionally ☐ Rarely ☐ Never ☐

F6. When a doctor/ health practitioner advises that I should take medicine three times a day, I know that I should take the medicine every 8 hours.

Yes ☐ No ☐ Not sure ☐

F7. Do you complete the full course of medication when prescribed for malaria treatment or any treatment, even if symptoms disappear?

Always ☐ Often ☐ Occasionally ☐ Rarely ☐ Never ☐

F8. If needed, I ask my doctor or health care practitioner questions about malaria or any illness.

Always ☐ Often ☐ Occasionally ☐ Rarely ☐ Never ☐
